# Enhanced Multifaceted Properties of Nanoscale Metallic Multilayer Composites

**DOI:** 10.3390/ma17164004

**Published:** 2024-08-12

**Authors:** Mahmoud Ebrahimi, Bangcai Luo, Qudong Wang, Shokouh Attarilar

**Affiliations:** 1 Department of Mechanical Engineering, Faculty of Engineering, University of Maragheh, Maragheh 83111-55181, Iran; ebrahimi@maragheh.ac.ir; 2 National Engineering Research Center of Light Alloy Net Forming and Key State Laboratory of Metal Matrix Composites, School of Material Science and Engineering, Shanghai Jiao Tong University, Shanghai 200240, China; sh.attarilar@yahoo.com; 3 Ningbo Major Draft Beer Equipment Co., Ltd., Ningbo 315033, China; boyce@chinesebrass.com; 4 Department of Materials Engineering, Faculty of Engineering, University of Maragheh, Maragheh 83111-55181, Iran

**Keywords:** nanoscale metallic multilayer materials, magnetic behavior, optical properties, radiation tolerance characteristics, thermal features, electrical resistance

## Abstract

This study explored the fascinating field of high-performance nanoscale metallic multilayer composites, focusing on their magnetic, optical, and radiation tolerance properties, as well as their thermal and electrical properties. In general, nanoscale metallic multilayer composites have a wide range of outstanding properties, which differ greatly from those observed in monolithic films. Their exceptional properties are primarily due to the large number of interfaces and nanoscale layer thicknesses. Through a comprehensive review of existing literature and experimental data, this paper highlights the remarkable performance enhancements achieved by the precise control of layer thicknesses and interfaces in these composites. Furthermore, it will discuss the underlying mechanisms responsible for their exceptional properties and provide insights into future research directions in this rapidly evolving field. Many studies have investigated these materials, focusing on their magnetic, mechanical, optical, or radiation-tolerance properties. This paper summarizes the findings in each area, including a description of the general attributes, the adopted synthesis methods, and the most common characterization techniques used. The paper also covers related experimental data, as well as existing and promising applications. The paper also covers other phenomena of interest, such as thermal stability studies, self-propagating reactions, and the progression from nanomultilayers to amorphous and/or crystalline alloys. Finally, the paper discusses challenges and future perspectives relating to nanomaterials. Overall, this paper is a valuable resource for researchers and engineers interested in harnessing the full potential of nanoscale metallic multilayer composites for advanced technological applications.

## 1. Introduction

Nanoscale metallic multilayers (NMMs) are materials composed of alternating nanoscale layers of two or more different homogenous materials [1,2]. They have been shown to have a wide range of exceptional properties that substantially vary from those reported for layered materials, mainly due to the existence of numerous interfaces and the nanoscale layer thicknesses. In general, NMMs and nanoscale metallic multilayer composites (NMMCs) have been found to have high strength, ductility, and toughness, as well as outstanding thermal and electrical conductivity, radiation tolerance, and optical and magnetic properties [1,3]. The exceptional properties of NMMCs make them attractive for a wide range of applications, including in the aerospace, automotive, and biomedical industries. However, the synthesis of NMMCs can be challenging, and the selection of a particular synthesis methodology depends on several factors, including growth rate/morphology, residual stresses, impurities, reproducibility, and microstructural imperfections, among others.

### 1.1. Outline of Multifunctional Properties

NMMCs have been shown to exhibit a wide range of magnetic properties, making them attractive for various applications. These materials exhibit the giant magnetoresistance (GMR) phenomenon, in which the electrical resistance of a material changes in the presence of a magnetic field [4]. This property has been exploited in the development of magnetic sensors, such as read heads for hard disk drives [4]. The NMMCs can also exhibit magnetic anisotropy, which is a directional dependence of the magnetic properties of a material [5]. This property has been used in the development of magnetic recording media, such as magnetic tapes and disks [5,6]. NMMCs are also capable of showing exchange bias, which is a phenomenon where the magnetic properties of a material are influenced by the presence of another magnetic material. This property has been used in the development of magnetic sensors and magnetic random access memory (MRAM) [5]. Additionally, NMMCs could have a magnetic shape memory characteristic, which is a phenomenon where the shape of a material changes in response to a magnetic field that is utilized in the development of microactuators and microsensors [1,7,8,9].

**Figure 1:**
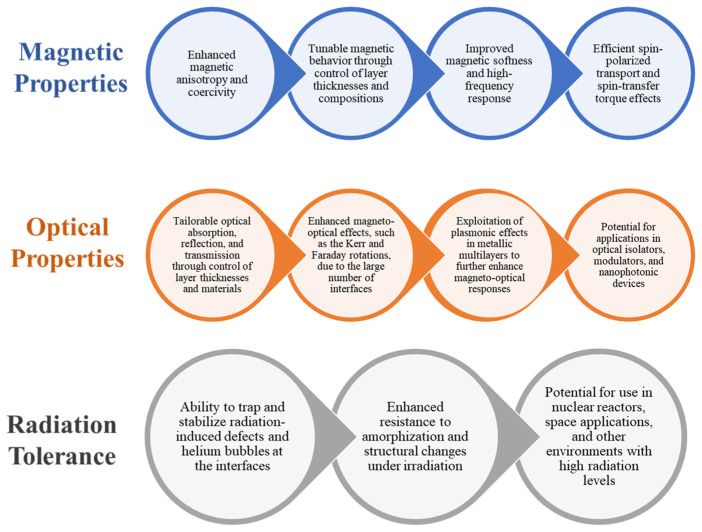
The evolution roadmap of nanoscale metallic multilayer composites (NMMCs).

NMMCs have been reported to exhibit unique optical properties, finding numerous varied applications. NMMCs can exhibit plasmon resonance, which is a phenomenon where the electrons in the metal layers oscillate in response to incident light, leading to enhanced light absorption and scattering [10]. This property has been used in the development of plasmonic sensors, surface-enhanced Raman spectroscopy (SERS), and photovoltaic devices [10]. They also display optical interference, which is a phenomenon where the light waves reflected from the different layers of the material interfere with each other, leading to enhanced or suppressed reflection and transmission. This property has been used in the development of optical filters, mirrors, and anti-reflection coatings [11]. Additionally, NMMCs have nonlinear optical properties, which are optical effects that are proportional to the square or higher powers of the incident light intensity. Nonlinear optical devices like frequency doublers and optical switches have developed using this property [10]. NMMCs have shown a wide range of exceptional magnetic, optical, and radiation tolerance properties that differ significantly from their monolithic counterparts. These properties can be broadly categorized into three main areas. In this regard, Figure 1 shows the NMMCs’ evolution roadmap.

Along with the unique properties of NMMCs, it should be noted that the size of the individual layers is highly relevant to their physical properties. The properties of NMMCs are strongly dependent on the thickness of the individual layers. As the layer thickness is reduced to the nanoscale, the materials exhibit size-dependent mechanical, electrical, thermal, and magnetic properties that significantly differ from their bulk counterparts [12]. For instance, the electrical conductivity and magnetoresistance of NMMCs can be tuned by controlling the layer thicknesses, leading to enhanced performance compared to monolithic films [1,13]. Moreover, the nanoscale dimensions and high density of interfaces in NMMCs enhance their thermal stability and ability to maintain properties at elevated temperatures [1,13]. Meanwhile, NMMCs can exhibit enhanced magnetic properties, such as increased magnetoresistance, compared to single-layer films, by leveraging the interfaces between magnetic and non-magnetic layers. In summary, the nanoscale dimensions of the individual layers in NMMCs are crucial in determining their unique and tunable physical properties, which differ significantly from bulk materials and enable their use in advanced technological applications. Additionally, the shape of the layers, whether planar, columnar, or other geometries, can also impact the properties of NMMCs. For example, columnar layer structures have been shown to exhibit improved mechanical strength and radiation tolerance compared to planar multilayers [1,14].

There are numerous applications for NMMCs in nuclear facilities and space exploration due to their exceptional radiation-tolerance properties. Here are some of the radiation-tolerance properties of NMMCs and their potential applications:

(i) Reducing the repeat layer spacing to the nanoscale results in an extremely high density of heterophase interfaces, enhancing radiation resistance and facilitating the development of ultra-high-strength V-graphene nanolayers for nuclear structural applications [15];
(ii) NMMCs can also be engineered to reduce radiation-induced crystalline defects, which leads to improved radiation tolerance, and they can be used in the production of amorphous/crystalline composites with greatly improved radiation tolerance [15];
(iii) The development of radiation-resistant materials for nuclear facilities and space exploration has used their enhanced mechanical properties, such as high strength and ductility, even after exposure to high levels of radiation [16,17].

Researchers have discovered that NMMCs exhibit exceptional thermal and electrical properties, which have led to numerous fascinating applications. Here are some of the thermal and electrical properties of NMMCs and their potential applications:

- In the case of thermal properties:
  (i) Thermal barrier coatings, heat exchangers, and other high-temperature components have been developed using NMMCs due to their excellent thermal stability [18].
  (ii) NMMCs have a high thermal conductivity, which makes them attractive for thermal management applications. They are used to produce thermal interface materials, heat sinks, and other thermal management devices [19].
- Regarding the electrical properties of NMMCs:
  (i) The development of electrical contacts, interconnects, and other electrical components can utilize their high electrical conductivity [1].
  (ii) NMMCs can be tailored to manifest specific electrical resistance values, which makes them ideal for various electrical applications, such as resistors, strain gauges, and other electrical components [1].

According to reports, NMMCs have many benefits, including enhanced multifunctional properties, tailored functionality, improved mechanical strength, and efficient energy conversion and storage, but they also have some drawbacks, including increased fabrication complexity, potential interfacial instability, a limited understanding of interfacial phenomena, and characterization challenges [13]. In summary, NMMs and NMMCs have gained significant attention in recent years due to their unique combination of properties and potential applications in various fields. Some of the studies conducted on the properties of metallic multilayers are summarized and listed in Table 1. This paper aims to provide an overview of the high-performance characteristics exhibited by these composites, focusing on their magnetic, optical, thermal, electrical, and radiation tolerance properties. These collections of exceptional properties make them outstanding candidates for various applications, but further research is needed to fully understand the varied properties of NMMCs, develop new applications for these materials, and utilize them in designing modern high-performance nanoscale metallic multilayer composites. It should be noted that this study builds upon previous publications in several key ways:

(i) It provides a comprehensive review of the existing literature on NMMCs, summarizing findings related to their magnetic, optical, radiation tolerance, thermal, and electrical properties.
(ii) The paper emphasizes how precisely controlling layer thicknesses and interfaces in these composites can achieve remarkable performance enhancements. This level of control allows for tuning their properties to a great extent.
(iii) It discusses the underlying mechanisms responsible for the exceptional properties of NMMCs, providing insights into future research directions in this rapidly evolving field.
(iv) This study covers other phenomena of interest, such as thermal stability studies, self-propagating reactions, and the progression from nanomultilayers to amorphous and/or crystalline alloys.
(v) It discusses challenges and future perspectives relating to the design and implementation of nanomaterials for advanced technological applications.

In this context, the current study not only explores the properties and applications of NMMCs but also analyzes the challenges and future prospects, thereby serving as a valuable resource for researchers and engineers who aim to fully utilize nanoscale metallic multilayer composites for advanced technological applications.

**Table 1:** Some of the studies conducted on the properties and key results of metallic multilayers.

Properties	Material	Method	Key Results	Ref.
**Magnetic**	Epitaxial symmetric (Fe/Ni)fcc^001^ multilayers	Sputter deposited	Enhanced magnetic coercivity by 30% compared to bulk materials	[20]
	Fe/Cu multilayers	Prepared under high vacuum conditions	Enhanced magnetic properties due to exchange coupling between layers	[21]
	Fe/Fe-N multilayered films	Sputter deposited	Improved soft magnetic properties	[22]
	Fe/Cu-N multilayered films	Sputter deposited	The oscillations of the in-plane saturation field, coercive force, and remanence ratio	[22]
	Cu/Fe multilayers	DC-magnetron sputtering	Change in magnetic behavior with the evolution from multilayer to island structures rather than the formation of a nonmagnetic FCC-Fe phase	[23]
	Fe-Ni/Cu	DC-magnetron sputtering	Fe-Ni/Cu multilayers exhibit alternate ferromagnetic and antiferromagnetic coupling	[24]
**Optical**	Au/Ag nanocomposite clusters	Thermal annealing	Tunable plasmonic response in the visible spectrum with high reflectance efficiency	[25]
	Ag/Au bilayer thin films	Electron beam deposition	By adjusting the mass–thickness ratio between Au and Ag, bilayer films’ spectral dispersion of the effective refractive index can be tuned	[26]
	A multilayer stack composite of alternating layers of Ag and TiO_2_	Sputtering	The transmission decreases as the number of multilayer pairs increases due to metal absorption	[27]
**Radiation tolerance**	Bulk nanolayered Cu/Nb composites	Accumulative roll bonding	Improved resistance to radiation-induced damage with minimal structural degradation	[28]
	Nanometric Cr/Ta multilayer	Physical vapor deposition	This coating showed an extremely high radiation tolerance	[29]
	Cu/V, Cu/Mo, Fe/W, and Al/Nb nanostructured metallic multilayers	Sputtering	He can be stored in extremely high concentrations in nanolayer composites; by encouraging the recombination of point defects of the opposite type, layer interfaces lessen lattice distortion, swelling, and accumulative defect density; interfaces also significantly reduce radiation hardening	[30]
**Thermal**	Al/Cu multilayer composite	Repeated hydrostatic extrusion process	High thermal conductivity and low coefficient of thermal expansion for efficient heat dissipation applications	[31]
	Multilayered Cu mesh/AZ31 Mg foil composites	Diffusion bonding	The α-Mg region and intermetallic compounds form a continuous film-like structure that contributes significantly to heat conduction, making it useful for designing and creating Mg matrix composites with high thermal conductivity	[32]
	Al/Cu laminated multilayered metal composites	Explosion welding and heat treatment	The thermal resistance of multilayered Al/Cu composites is enhanced by thin Cu layers and a high-volume fraction of intermetallics	[33]
	Multilayered Al/Cu metal matrix composite	Cold roll bonding and accumulative roll bonding	The thermal conductivity from the Al layer to the Cu layer increased with an increase in copper content, demonstrating the good conductance of the Al/Cu interface and copper’s constituent parts	[34]
**Electrical**	Cu/Nb multilayer	Sputtering	Enhanced electrical conductivity attributed to electron scattering at interfaces	[35]
	Multilayered metallic thin films	Sputtering	Electrical conductivity due to their enhanced interactions at the interfaces between different metals	[36]
	Ni/Pt and Co/Au_25_Cu_75_ multilayered system	Sputtering	These multilayers show a similar type of anomaly in electrical resistivity near a certain temperature, with a deep minimum in dρ/dT, reversible under temperature cycling	[37]
	Cu/Nb composites with continuous laminated structure	Accumulative roll bonding	The nanolaminated Cu/Nb composites retained excellent electrical conductivity	[38]

### 1.2. Motivation

The field of materials science and engineering has witnessed significant advancements in recent years, particularly in the development of nanoscale metallic multilayer composites [13]. These composites, consisting of alternating layers of different metallic materials at the nanoscale, have shown exceptional promise in various applications due to their unique combination of magnetic, optical, thermal, electrical, and radiation-tolerance properties. One key motivation for studying high-performance nanoscale metallic multilayer composites is their potential to revolutionize the fields of electronics, photonics, and energy conversion. The ability to tailor the properties of these composites at the atomic level opens up new possibilities for designing advanced devices with improved performance and efficiency [39,40]. For instance, by carefully selecting the constituent materials and layer thicknesses, one can achieve enhanced magnetic properties for applications in data storage or sensing technologies. Similarly, precise control over optical properties allows for the development of novel photonic devices with improved light absorption or emission characteristics. Another important motivation lies in the quest for materials that can withstand extreme conditions. NMMCs have demonstrated remarkable resistance to radiation damage, making them highly suitable for applications in nuclear reactors or space exploration, where exposure to high-energy particles is a concern. Their ability to maintain structural integrity and functionality in harsh environments makes them attractive candidates for next-generation materials that can withstand extreme temperatures or corrosive atmospheres [41,42,43].

Furthermore, these composites offer intriguing opportunities for improving thermal management in electronic devices. By exploiting their unique thermal conductivity properties, it becomes possible to design more efficient heat sinks or thermal interface materials that can dissipate heat effectively and prevent device failure due to overheating. This aspect is particularly relevant as electronic devices continue to shrink in size while becoming more powerful. In summary, exploring the magnetic, optical, thermal, electrical, and radiation tolerance properties of high-performance nanoscale metallic multilayer composites holds great promise for advancing various technological fields. The ability to tailor their properties at the atomic scale opens up new avenues for developing advanced devices with improved performance and durability. By understanding and harnessing these unique characteristics, researchers can pave the way toward innovative applications that will shape our future technology landscape [44,45].

## 2. Magnetic NMMCs

NMMCs exhibit unique magnetic properties due to their specific structure and composition. These composites consist of alternating layers of different metallic materials, typically with layer thicknesses ranging from a few nanometers to tens of nanometers. Various factors, including the type of metal used, layer thicknesses, interface quality, and the presence of interdiffusion or intermixing between layers, influence the magnetic properties of these composites [46,47].

The giant magnetoresistance effect is an important magnetic property observed in nanoscale metallic multilayer composites. GMR refers to the significant change in electrical resistance that occurs when an external magnetic field is applied parallel or antiparallel to the direction of electron flow. This effect arises due to the spin-dependent scattering of electrons at the interfaces between different metallic layers [48,49]. The GMR effect has been extensively studied and utilized in various applications, such as magnetic sensors, read heads in hard disk drives, and non-volatile memory devices [49]. Exchange coupling is another interesting magnetic property exhibited by these composites. Exchange coupling refers to the interaction between adjacent magnetic layers through their shared interfaces. In NMMCs, exchange coupling can be either ferromagnetic or antiferromagnetic, depending on the relative orientation of magnetization between adjacent layers [50]. Ferromagnetic exchange coupling leads to a parallel alignment of magnetization, while antiferromagnetic exchange coupling results in an antiparallel alignment. The presence of exchange coupling can significantly affect the overall magnetic behavior of these composites. For instance, it can enhance or suppress magnetic properties such as coercivity (the resistance to demagnetization), remanence (the residual magnetization after removing an external field), and saturation magnetization (the maximum attainable magnetization) [51,52,53]. By carefully engineering the layer thicknesses and materials used in NMMCs, it is possible to control and optimize these magnetic properties for specific applications. For example, a study reported a simple and scalable synthesis route to produce a magnetic exchange-coupled composite consisting of magnetically hard SrFe_12_O_19_ and magnetically soft La_0.7_Sr_0.3_MnO_3_. The study found that the composite exhibited enhanced magnetic properties due to the exchange coupling between the two phases [54]. Another study demonstrated the correlation between composition, magnetic, optical, and microwave properties of exchange-coupled hard/soft magnetic nanocomposites [55,56].

**Figure 2:**
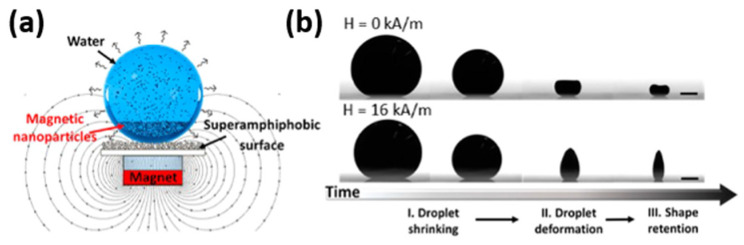
Superparamagnetic supraparticles. (**a**) The experimental setup for the evaporation-guided assembly of a magnetic nanoparticle dispersion on a superamphiphobic surface that produces supraparticles, and (**b**) the drying process of a 3% weight droplet in the absence (**upper** panel) and presence (**bottom** panel) of a magnetic field. Note that a 0.5 mm scale bar is used [57].

Furthermore, nanoscale metallic multilayer composites also exhibit superparamagnetic behavior at certain layer thicknesses. Superparamagnetism refers to a phenomenon where individual nanoparticles within a composite behave like independent superparamagnetic entities rather than exhibiting collective magnetism. This behavior arises due to thermal fluctuations overcoming the energy barrier required for maintaining a stable magnetization direction [57,58,59,60,61,62]. In an interesting study, Hu et al. [57] employed evaporation-guided assembly to dry ferrofluid droplets on a superamphiphobic substrate in the presence of an external magnetic field, resulting in the production of superparamagnetic supraparticles, as shown in Figure 2. Barrell-like, cone-like, and two-tower-like supraparticles were produced by adjusting the concentration of ferrofluid droplets and regulating the magnetic field. These supraparticles retained the superparamagnetism of the original nanoparticles [63]. In summary, NMMCs possess unique magnetic characteristics; these properties make them attractive for various technological applications, including data storage devices, sensors, and spintronics. Ongoing research continues to explore new material combinations and fabrication techniques to further enhance their magnetic performance and expand their potential applications.

### 2.1. Magnetism at the Nanoscale

At the nanoscale, magnetism in multilayer metallic composites can exhibit unique properties and behaviors. These composites typically consist of alternating layers of different metallic materials, each with their own magnetic properties. One important phenomenon observed at the nanoscale is the exchange coupling effect. The exchange coupling can result in enhanced or suppressed magnetization, depending on the specific configuration and thickness of the layers. Exchange interaction provides an efficient way to enhance the magnetic properties of nanostructured materials [64,65]. Exchange-coupled magnetic composites utilize this effect, with the synergistic action of constituent phases governing their properties. One of the issues that must be resolved before such composites are employed in industry is texturing [66]. A study investigated the influence of texture on the magnetic properties of anisotropic Co/Co_3_O_4_ exchange-bias composites based on nanorod arrays. The study found that the magnetic properties of such materials are dependent on their internal texture. Texture tailoring can lead to an increase in the exchange polarization field or an improvement in the material’s coercivity [52].

**Figure 3:**
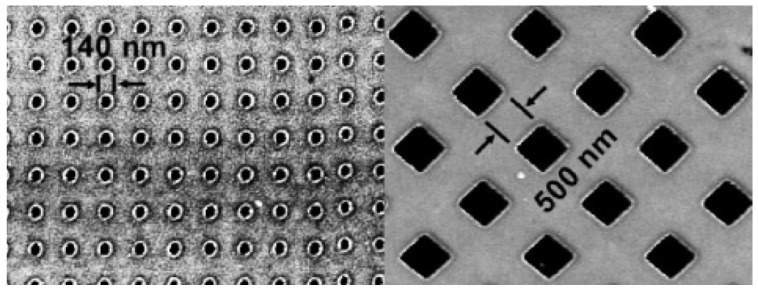
AFM image showing magnetic islands with diameters of 140 and 500 nm within a nonmagnetic matrix following patterning. Note that the Ar^+^ implantation has caused the matrix regions to enlarge by around 10 nm in height relative to the protected islands [69].

At the nanoscale, magnetic domain structures are an interesting aspect of nanoscale multilayer metallic composites. Factors such as layer thickness, composition, and interface quality can significantly influence these structures. The manipulation and control of these domain structures are critical for a variety of nanomagnetism applications. Using phase-field simulations, a study investigated the strain effects on magnetic domain stability and dynamics in nanoscale magnetic thin films [67]. The study found that the strain can be used to engineer domain structures in nanoscale magnetic thin films [68]. Another study synthesized highly spin-polarized complex magnetic oxide nanostructures embedded in a paramagnetic matrix by tuning the magnetic domain structure in nanoscale La_0.7_Sr_0.3_MnO_3_ islands, as shown in Figure 3 [69]. It was known that through careful choice of the hexagon aspect ratio, crystalline direction, and substrate orientation, the magnetic domain formation can be tuned to be single [70]. The study incorporated the highly spin-polarized material La_0.7_Sr_0.3_MnO_3_ into spin-based electronic devices, revealing enhanced spin-dependent transport properties in the resulting magnetic oxide materials. 

**Figure 4:**
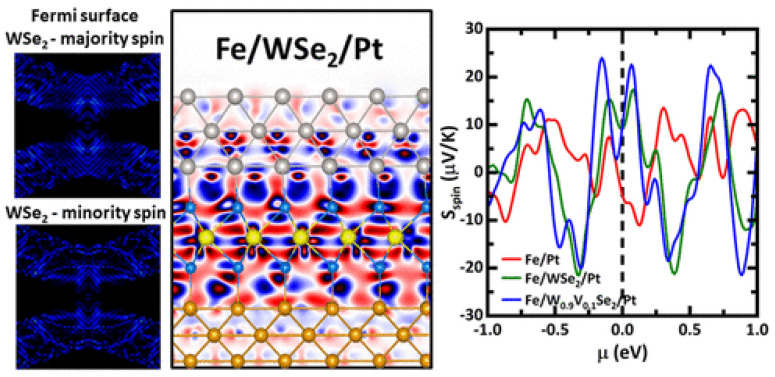
Unfolded Fermi surface WSe_2_ monolayer from the Fe/WSe2/Pt heterostructure along with its structure and spin Seebeck coefficient (*S*_spin_) as a function of chemical potential (*μ*) [71].

Two-dimensional materials and their heterostructures have opened up new possibilities for magnetism at the nanoscale. A study demonstrated enhanced magnetism in heterostructures with transition-metal dichalcogenide monolayers [71]. They examined the structural, electrical, and magnetic characteristics of Fe/WSe_2_/Pt systems with pristine, defective, or doped WSe_2_ monolayers using first-principles simulations, as shown in Figure 4. It has been discovered that all heterostructures are ferromagnetic, and the magnetized WSe_2_ causes a redistribution of spin orientation and an increase in the density of magnetic atoms upon the insertion of the transition-metal dichalcogenide. WSe_2_ increases the total density of states at the Fermi level, but greater than van der Waals coupling may cause the transition-metal dichalcogenide to lose its unique semiconducting characteristics. Larger spin Seebeck values observed in heterostructures containing WSe_2_ monolayers are associated with spin-resolved electronic structural characteristics. The study found that the magnetism in the heterostructures was enhanced due to the interfacial coupling between the transition-metal dichalcogenide monolayers and the magnetic layer. The enhanced magnetism in these heterostructures has potential applications in spintronics, magnetic storage, and magnetic sensors [71].

The magnetoelectric effect is the coupling between electric and magnetic fields, making it possible to modulate an electric polarization with a magnetic field. A study investigated the nanoscale domain structure evolution and magnetoelectric coupling for the PMN-33PT/Terfenol-D multiferroic composite [72]. The study found that the composite PMN-33PT/Terfenol-D shows stronger ME coupling than that of PMN-28PT/Terfenol-D [73,74]. The study also reported the magnetic-field-induced ferroelectric polarization reversal in the bilayer Terfenol-D/PMN-33PT composite [73]. The finer domain band structure and more domain variants contribute to the stronger piezoelectric activity. The study highlights the potential of multiferroic composites for applications in sensors, actuators, and energy-harvesting devices. 

In this review article, domain structure visualization and other characterization techniques are discussed for their application in materials science and biomedicine. The article highlights the importance of understanding magnetic domains and their structures for various applications [75]. Furthermore, nanoscale multilayer metallic composites can exhibit magnetoresistance effects. Magnetoresistance is defined as changes in electrical resistance caused by an applied magnetic field. In these composites, variations in resistance occur as a result of changes in electron scattering at interfaces between different magnetic layers. Overall, studying magnetism at the nanoscale in multilayer metallic composites provides valuable insights into fundamental physics and offers potential for developing advanced technologies such as high-density data storage devices, spintronics, and sensors [76,77,78,79].

### 2.2. Magnetic Multilayer Composites

Magnetic multilayer composites are structures composed of multiple layers of magnetic and non-magnetic materials [80]. These composites have a wide range of applications in various fields, including spintronics, magnetic storage, and integrated RF passive devices (electrical integrated circuits that operate in the radio frequency range) [81]. The thickness of each layer in magnetic multilayers can be controlled to achieve specific magnetic properties. DMI refers to the Dzyaloshinskii–Moriya interaction. This interaction is a phenomenon that occurs at the interfaces between magnetic layers in a multilayer structure, leading to specific magnetic properties and behaviors [80]. Magnetic multilayer systems exhibiting interfacial DMI typically embed the magnetic layers below several other layers and cover them with a non-magnetic layer [80]. Researchers have studied the magnetoelectric effect in a double-layered structure based on a piezoelectric polymer and a magnetoactive elastomer with iron microparticles, revealing dynamic control of the magnetoelectric effect in the composite structure [82]. The study investigated the double-layered structure based on the piezoelectric polymer polyvinylidene fluoride and a magnetoactive elastomer with iron microparticles. When this structure was subjected to a gradient AC magnetic field, the sample bent as a result of the magnetic component’s attraction. It was noted that the magnetoelectric effect was resonantly enhanced. The primary resonance frequency of the samples was determined by the magnetoactive elastomer (MAE) characteristics, specifically the iron particle concentration and thickness, and ranged from 50–72 Hz for a 3 mm MAE layer to 156–163 Hz for a 0.3 mm MAE layer. The resonant frequency was also dependent on the bias DC magnetic field [82].

**Figure 5:**
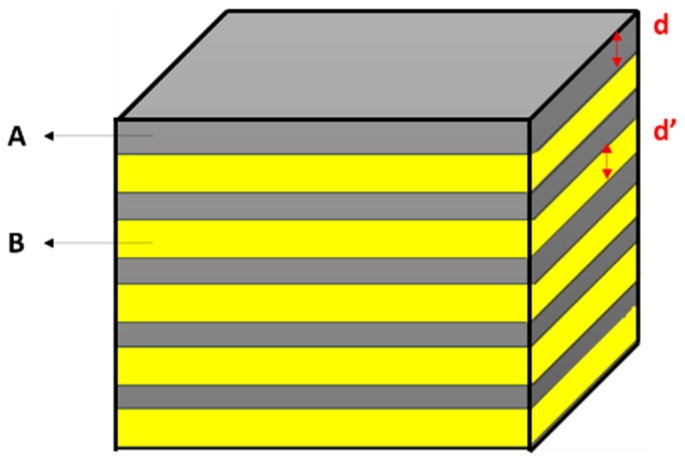
Diagram showing ferromagnetic A and non-magnetic B layers in magnetic multilayers. Note that d and d′ indicate the layer’s thickness.

It should be noted that magnetic metallic multilayers are a multilayer film system resulting from the alternately repeated growth of nanoscale ferromagnetic films (Fe, Co, Ni, and their alloys) [83]. The basic structure of magnetic multilayers composed of a ferromagnetic metal and a non-magnetic metal is shown in Figure 5. For instance, FeRh is a naturally occurring magnetic multilayer structure that shows giant changes in magnetoresistance [84]. 

## 3. Optical NMMCs

**Figure 6:**
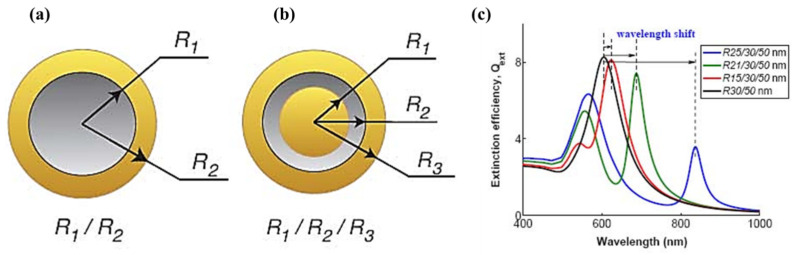
The schematic representation of (**a**) conventional silica–gold core nanoshells, (**b**) multilayer gold–silica–gold nanoshells, and (**c**) conventional silica–gold core and multilayer gold–silica–gold nanoshell calculated spectra with different inner core radii but the silica and outer radii staying the same (Media 1). Note that the lambda shift indicates the multilayer gold–silica–gold nanoshells’ red shift from the conventional silica–gold core nanoshells [85].

Numerous investigations have also focused on the optical properties of nanoscale metallic multilayer composites, such as nanoshells [85], metal-dielectric composite-based multilayers [86], nanocomposites [87], and van der Waals heterostructures [88]. A study investigated the optical properties of gold-silica-gold multilayer nanoshells and found that multilayer nanoshells are more absorbent with a larger gold core, a thinner silica layer, and a thinner outer gold shell [85]. Researchers discovered that the inner core’s spectrum modulation caused differences in the scattering intensity and angular radiation pattern compared to ordinary nanoshells. At wavelengths where silica-gold core-shell nanoshells primarily scatter forward, multilayer nanoshells might offer more backscattering. Figure 6 represents the schematics of conventional (Figure 6a) and multilayer silica-gold core nanoshells (Figure 6b). Furthermore, the thickness of the intermediate silica layer will decrease, and the plasmon interaction will grow as the inner gold core radius increases on an otherwise unchanged shape. This is accompanied by a spectrum redshift, as seen in Figure 6c [85]. It was found that due to the spectrum modulation caused by the core, multilayer nanoshell scattering patterns are different from conventional nanoshell scattering patterns. Compared to conventional multilayer nanoshells, angular radiation patterns exhibit more complex trends. At wavelengths less than the plasmon resonant wavelength, multilayer nanoshells may radiate more in the side and back directions, whereas conventional nanoshells mostly scatter forward [85].

Another study prepared metal-dielectric composite-based multilayer thin-film structures using radio frequency (RF)-magnetron sputtering deposition and reported on their optical and chromaticity properties compared to pure metal-based dielectric/metal/dielectric (DMD) trilayer structures with similar compositions [86]. It was confirmed that metal-dielectric composite (MDC) layers can provide greater flexibility in the design of structured optical coatings with potentially better visible color tunability, which is important for many modern industrial and commercial applications. They reported that the development of Ag:MgF_2_ MDCs was expected to achieve the following results:

(i) Promote the adhesion of metal layers to their substrates;
(ii) Increase the mechanical hardness of soft plasmonic metal layers;
(iii) Improve the morphology of metal layers by “filling the pores” between the ultrathin metal nanoislands;
(iv) Potentially smoothing the interface roughness of the ultrathin metal layers [86].

**Figure 7:**
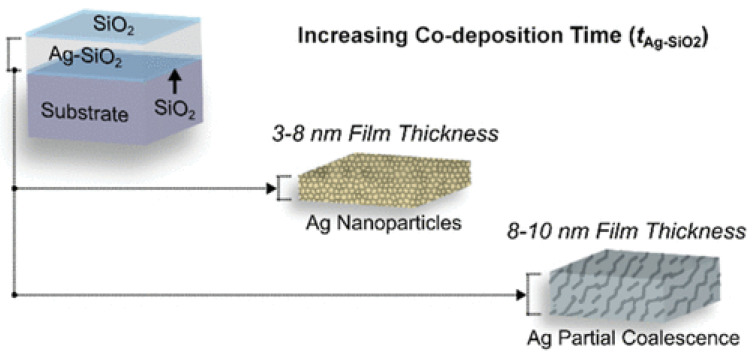
The structural characteristics of nanocomposite Ag-SiO_2_ films and the effect of altering co-sputtering duration at film thicknesses [87].

A third study demonstrated that varying co-sputtering can manipulate the structural features and optical properties of nanocomposite Ag-SiO_2_ films [87]. When compared to traditional all-dielectric multilayer optical media, co-sputtering of nanocomposite metal–dielectric films presents an enticing path toward ultrathin film coalescence and customizable optical properties to achieve high-precision optical performance at significantly reduced film thicknesses. In the study of Sun et al. [87], through magnetron co-sputtering in a regulated pure argon atmosphere, silicon dioxide (SiO_2_) was used to create contiguous silver (Ag) networks and Ag nanoparticles embedded in the material at room temperature, as shown in Figure 7. It was observed that by altering the co-sputtering duration at film thicknesses of approximately 3–10 nm, the structural characteristics and optical attributes of nanocomposite Ag-SiO_2_ films can be controlled. After Ag phases transition from nanoparticles to the partially coalesced network, a clear optical response transition takes place. At these reduced film thicknesses, significant variations in the measured optical intensity are seen: maximum ΔT = 67%, ΔR = 28%, and ΔA = 46% in the visible and near-infrared regions. It was demonstrated that optimizing the refractive index (*n*) and extinction coefficient (*k*) of ultrathin-metal-film optical materials is anticipated to yield workable approaches for the creation, deposition, and integration of next-generation complex index multilayer optical filters and mirrors with improved precision spectral performance [87].

Ramos et al. [88] studied the properties of a specific van der Waals heterostructure composed of multilayer FePS_3_ (p-type) and single-layer MoS_2_ (n-type). The FePS_3_/MoS_2_ heterostructure demonstrates a tunable, multifunctional opto-electrical response in which robust photoluminescent light emission and broadband electrical photo-response coexist. This is enabled by the inherent properties of the materials and the precise energy band alignment at their interface. Specifically, small changes in the applied voltage across the junction allow for precise control of the light emission of single-layer MoS_2_, ranging from severely quenched to an order of magnitude enhancement. These findings demonstrate the versatility and additional functionality enabled by the combination of specific van der Waals semiconductors in vertical stacks, leading to atomically sharp heterointerfaces with unique properties for thin, flexible optoelectronic devices [88].

### 3.1. Light–Matter Interaction at the Nanoscale

**Figure 8:**
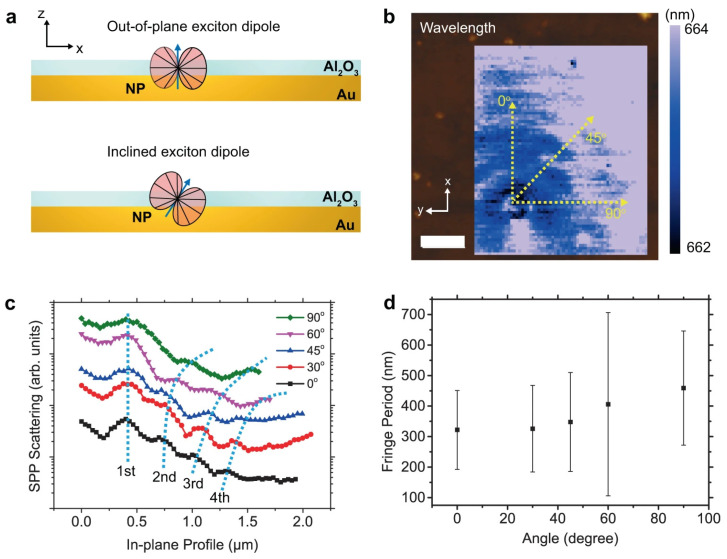
Analysis of fringe patterns: (**a**) the diagram illustrates the exciton transition dipole’s schematic representation, with the top figure showing its out-of-plane orientation and the bottom figure demonstrating its inclined angle; (**b**) the near-field emission wavelength map of Al_2_O_3_ (5 nm)/NP/Au is displayed with a topography background; (**c**) the line-cuts of TEPL intensity at various direction angles; and (**d**) the fringe period expressed mathematically as a function of the angle. Note that the error bar corresponds to the full width at half maximum (FWHM) of the peak observed in the Fourier transform of the fringe profiles in (**c**). The scale bar represents 0.5 µm [91].

In metallic multilayers, light–matter interaction at the nanoscale is a growing field of research known as nano-optics and nano-photonics [89]. Many studies have investigated the interaction between light and matter at the nanoscale, including the control of this interaction and its possible applications. The optical properties of nanoscale metallic multilayer composites have also been studied [1,90,91]. The exceptional properties of NMMCs are mainly associated with the large number of interfaces and the nanoscale layer thicknesses [1]. The following are some of the studies that have investigated light–matter interaction at the nanoscale in metallic multilayers. A recent study reported direct nano-imaging of light–matter interactions in nanoscale excitonic emitters and found that understanding light–matter interactions in materials with strongly resonant properties and deep-subwavelength dimensions is important for both basic science and nano-opto-electronic applications [91]. In this regard, Jo et al. [91] investigated a near-field nano-spectroscopic phenomenon in localized nanoscale emitters on a flat Au substrate via quasi-two-dimensional CdSe/Cd_x_Zn_1−x_S nanoplatelets (NPs). They located directional propagation at the Au substrate of surface plasmon polaritons released from the excitons of the nanoplatelets as wave-like fringe styles inside the near-field photoluminescence maps. These fringe patterns have been shown through great electromagnetic wave simulations to be standing waves shaped among the end, and the edge-up assembled nano-emitters at the substrate plane. In this regard, Figure 8 shows the analysis of fringe patterns; fringe styles are best seen for thick NP clusters (>16 nm), whereas they are no longer seen for skinny NP clusters (~3 nm). It was also seen that edge-up NP clusters release surface plasmon polaritons (SPPs), whereas the alternative does not. Therefore, changing the polarization of the excitation laser is a much better way to swap the SPPs that are released from edge-up assembled NP clusters. Note that the difference in excitation energy and exciton emission energy significantly influences the mechanism responsible for fringe creation [91].

### 3.2. Plasmonic Effects in Multilayer Composites

Plasmonic effects in metallic multilayers refer to the interaction between light and free electrons in metals, which can result in the confinement of electromagnetic fields to subwavelength dimensions. In this regard, these subjects should be carefully discussed:

(i) Plasmon coupling and finite size effects [92,93];
(ii) Electromagnetically induced transparency (EIT) effect [94,95];
(iii) Fano resonance and giant field enhancement [96];
(iv) Plasmon-enhanced light–matter interactions [97,98]; and
(v) Multilayer plasmonic nanostructures for improved sensing activities [99,100,101].

In the first case, a study used electron energy loss spectroscopy (EELS) to investigate the excitation of interface and bulk plasmons in artificial, nonmagnetic metallic multilayers [92]. The results showed that the plasmon coupling and finite size effects can significantly affect the plasmon dispersion and damping in these systems. In the case of the EIT effect, another study reported the observation of EIT in a plasmonic metamaterial light absorber based on multilayered metallic nanoparticle sheets [94]. The Ag nanosheets used in this study can act as a plasmonic metamaterial light absorber, which has a large oscillator strength. This new type of EIT effect using a plasmonic metamaterial light absorber presents the potential for the development of future optic and photonic technologies. In the third case, a review article provided an overview of Fano resonance and plasmon-induced transparency (PIT), as well as plasmon coupling in planar structures [96]. The article also discussed the plasmonic coupled modes in metal-dielectric multilayer structures, which can result in Fano resonance and giant field enhancement. Considering plasmon-enhanced light–matter interactions, it should be stated that plasmons can greatly accumulate the optical field and energy on the nanoscale and dramatically enhance various light–matter interactions [97]. The properties of surface plasmons are strongly related to materials and structures, so metals, semiconductors, and two-dimensional materials with various morphologies and structures can have alternating plasmonic wavelengths ranging from ultraviolet, visible, and near-infrared to far-infrared. Lastly, a recent study proposed a multilayer plasmonic nanostructure for improved sensing activities using a finite element method (FEM) and a neurocomputing-based approach [99]. The study showed that the proposed multilayer plasmonic nanostructure can significantly enhance the sensitivity and selectivity of sensing activities.

**Figure 9:**
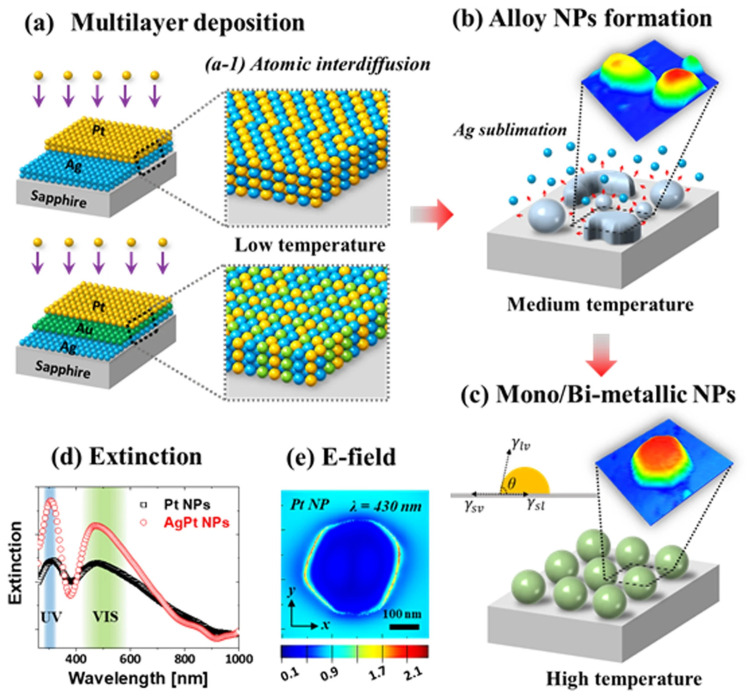
Production of monometallic and multimetallic alloy nanoparticles, as well as the simultaneous effects of sublimation, dewetting, and interdiffusion. (**a**) Diagrams showing how Ag/Pt and Ag/Au/Pt multilayers are deposited on sapphire (0001) (atomic diffusion at low temperatures during annealing (a-1)), (**b**) sublimation of Ag atoms while alloy nanoparticles (NPs) are formed, (**c**) Pt and AuPt NP formation following Ag sublimation, (**d**) extinction rates of common AgPt and Pt NPs are compared, and (**e**) local e-field distribution using finite difference time domain (FDTD) simulation of a typical Pt NP [102].

In the study of Sui et al. [102], the fabrication process included the creation of different types of nanostructures composed of Ag, Au, and Pt on transparent c-plane sapphire (0001) substrates. These structures were carefully examined to understand their morphological and optical properties in detail. The Pt and AuPt nanoparticles obtained in this study show considerably improved localized surface plasmon resonance (LSPR) responses compared to the pure Pt nanoparticles from previous studies. This enhancement is attributed to the synergistic effect of Au and Pt, as well as improved surface morphology. The results obtained from the dewetting of bi-layer and tri-layer films exhibit significant differences in surface morphology and elemental variability compared to the dewetting of monometallic Ag, Au, and Pt films under similar growth conditions. This can be attributed to the unique dewetting kinetics of the bi-layer and tri-layer films. Depending on their size and elemental makeup, these NPs show significantly enhanced LSPR bands in the UV-VIS range, including dipolar, quadrupolar, multipolar, and higher-order resonance modes. With a high Ag content, the LSPR bands are substantially stronger, and as the Ag is sublimated, they gradually become weaker. Additionally, when the size of the NP decreases, the LSPR bands in the VIS region are easily blue-shifted. By using enhanced surface diffusion, intermixing, energy minimization, and temperature control, the Ag/Pt bi-layers and Ag/Au/Pt tri-layers are systematically dewetted and transformed into various AgPt and AgAuPt nanostructures, such as networked, elongated, and semispherical configurations. Ag atom sublimation has a major effect on the structural and elemental makeup of NPs, leading to the evolution of more isolated and semispherical Pt and AuPt NPs from AgPt and AgAuPt NPs, respectively [102]. In this regard, the systematic annealing control of Ag/Pt bi-layers and Ag/Au/Pt tri-layer films that have been deposited is depicted in Figure 9, leading to different AgPt and AgAuPt alloy nanostructures in terms of size, shape, density, and elemental composition. The formation of alloyed NPs is controlled by temperature-induced surface diffusion and interdiffusion of atoms, nucleation, surface energy minimization, and equilibrium crystal structure. Moreover, the evolution of the NPs’ elemental composition and surface appearance, which produces Pt and AuPt NPs at high temperatures, is significantly influenced by the sublimation of Ag [102].

**Figure 10:**
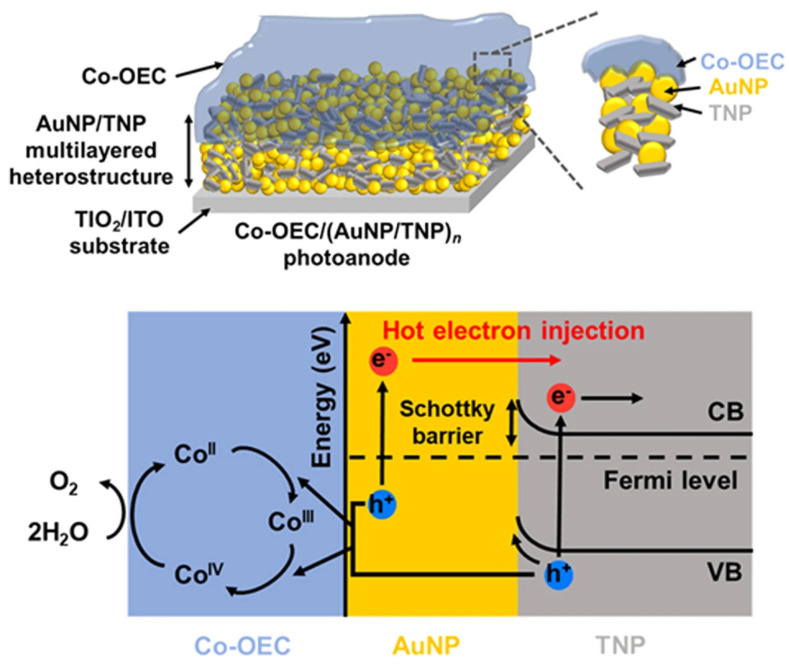
Schematic representation of the operating principle of the Co-OEC/(AuNP/TNP)n photoanode [103].

In another study, Kim et al. [103] used the LSPR technique to create a strong, multilayered plasmonic heterostructure for water oxidation by employing gold nanoparticles (AuNPs) as light-harvesting materials. The multilayered heterostructure is created by AuNPs and TiO_2_ nanoparticles (TNPs) self-assembling layer by layer. Charge separation of hot carriers occurs when plasmon-induced hot electrons cross the Au/TiO_2_ Schottky barrier and go from AuNPs to TNPs. A Co-based oxygen-evolving catalyst is added to the multilayered heterostructure of plasmonic photoanodes for water oxidation in order to scavenge hot holes. The photoanodes’ PEC characteristics and light absorption capacity are examined in relation to the quantity of AuNP/TNP bilayers present. Due to charge transport within the multilayered heterostructures, the PEC properties show dependence on the number of bilayers. Considerable photoactivity by LSPR excitation is indicated by the photocurrent density and the decrease in resistance to radiation. Figure 10 describes the operational principles of the photoanode that is being proposed in this work. After crossing the Schottky barrier, the hot electrons travel to the TiO_2_’s CB. When exposed to solar radiation, photoexcited electrons from the TiO_2_’s valence band (VB) to CB contribute to the photocurrent, which in turn drives the reduction reaction. Meanwhile, heated holes are moved from AuNPs to the Co-OEC interface, where they fuel the reaction that oxidizes water [103].

## 4. Thermal NMMCs

Studies have demonstrated a wide range of outstanding properties in NMMCs, such as thermal stability and high-temperature performance. In general, the thermal properties of nanoscale metallic multilayer composites are strongly influenced by the presence of interfaces and grain boundaries that can scatter phonons and affect the thermal conductivity of the material. The addition of metallic fillers or graphene can enhance the thermal conductivity of the composite. This section discusses some key findings from the research on the thermal properties of nanoscale metallic multilayer composites.

### 4.1. Thermal Conductivity in Multilayer Composites

**Figure 11:**
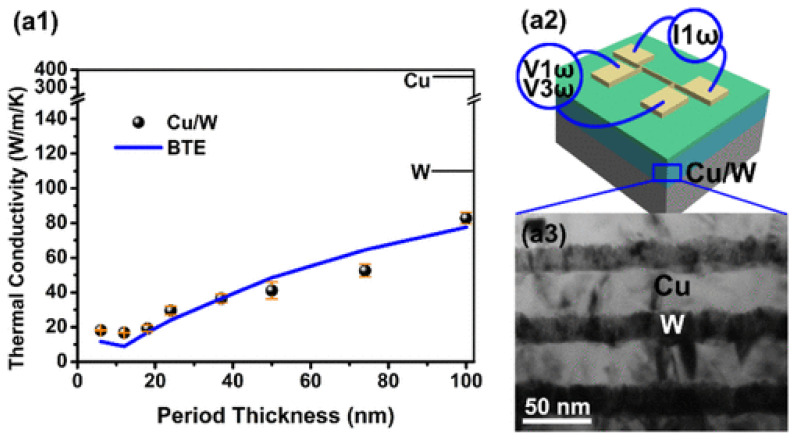
The thermal conductivity of Cu/W multilayered nanofilms at room temperature with varying periodic thicknesses, along with bright-field cross-sectional TEM images of the Cu/W multilayered nanofilms: (**a1**) thermal conductivity of Cu/W multilayered nanofilms curves at room temperature according to varied periodic thicknesses; (**a2**) structure of Cu/W multilayered nanofilms; (**a3**) bright-field cross-sectional TEM micrographs of the Cu/W multilayered nanofilms [106].

Enhancing thermal transport in layered nanomaterials presents a comprehensive rational thermal material design paradigm that requires the ability to reduce and enhance the thermal conductivities of nanomaterials [104]. It was known that, through nanoscale engineering, thermal conductivities could be enhanced. Several factors affect the thermal conductivity of NMMCs, including layer thickness, interface quality, composition, the phonon–glass effect, and surface electromagnetic waves. As the layer thickness decreases, the thermal conductivity decreases due to increased phonon scattering at the interfaces between the layers [105]. The quality of the interfaces between the layers in NMMCs affects the thermal conductivity. A high-quality interface can enhance thermal conductivity by reducing phonon scattering [106]. In the study of Dong et al. [106], Cu/W multilayered nanofilms were deposited using magnetron sputtering, and their periodic thickness ranged from 6 to 150 nm. The three-omega (3ω) method was used to measure the Cu/W multilayered nanofilms’ thermal conductivities. It shows that the thermal conductivities initially decrease with a decrease in periodic thickness and increase at the smallest periodic thickness of 6 nm. By taking into account the contributions from both phonon and electron heat transport processes, the Boltzmann transport equation (BTE)-based model was utilized to explain the periodic thickness-dependent thermal conductivity of metallic multilayered nanofilms, where the calculated thermal conductivities agree well with the measured ones [106]. In this regard, Figure 11 shows the thermal conductivity of Cu/W multilayered nanofilms at room temperature with varying periodic thicknesses, as well as bright-field cross-sectional TEM images of the Cu/W multilayered nanofilms [106].

It should be noted that different metallic compositions lead to different thermal conductivities, and the choice of metals can affect the overall thermal conductivity of the multilayer [1]. Also, NMMCs can be designed to have phonon–glass thermal conductivity, which is lower than that of bulk materials. This is due to the increased phonon scattering at the interfaces between the layers [107]. Furthermore, thermal-conductivity enhancement by surface electromagnetic waves propagating along multilayered structures with asymmetric surrounding media can lead to enhancement of thermal conductivity via surface electromagnetic waves (SEWs) supported in nanostructures as a remedy for issues raised due to the reduction of thermal conductivity in nanoscale confinement [108]. Among them, multilayered structures on a substrate are prevalent in nanosized systems, such as electronic nanodevices, meaning that the analysis of those structures is indispensable. In the study of Lim et al. [108], three basic multilayered structures are selected, and the analytical expressions for SEWs supported in each structure are derived. This analytical approach can be used to figure out which factors are crucial for enhancing SEW thermal conductivity using multilayers. It was also found that the solution can be extended to various materials, and guidelines were provided on which configurations are desirable for increasing thermal conductivity.

NMMCs have unique thermal conductivity properties that differ from those of other materials. The thermal conductivity of bulk materials is generally higher than that of NMMCs. This is because the thermal conductivity of bulk materials is mainly determined by the lattice vibrations, which are less affected by the size of the material [105]. Also, the thermal conductivity of thin films is lower than that of bulk materials, and it decreases further as the film thickness decreases. This is due to the increased scattering of phonons at the film interfaces and surfaces. Furthermore, the thermal conductivity of nanoparticles is lower than that of bulk materials, and it decreases further as the particle size decreases. This is due to the increased surface area-to-volume ratio, which leads to increased phonon scattering at the particle surfaces [105]. The thermal conductivity of nanowires is also lower than that of bulk materials, and it decreases further as the wire diameter decreases. This is due to the increased surface-to-volume ratio, which leads to increased phonon scattering at the wire surfaces. Finally, NMMCs can be designed to have phonon–glass thermal conductivity, which is lower than that of bulk materials. This is due to the increased phonon scattering at the interfaces between the layers [107].

### 4.2. Transport Mechanisms of Phonon

**Figure 12:**
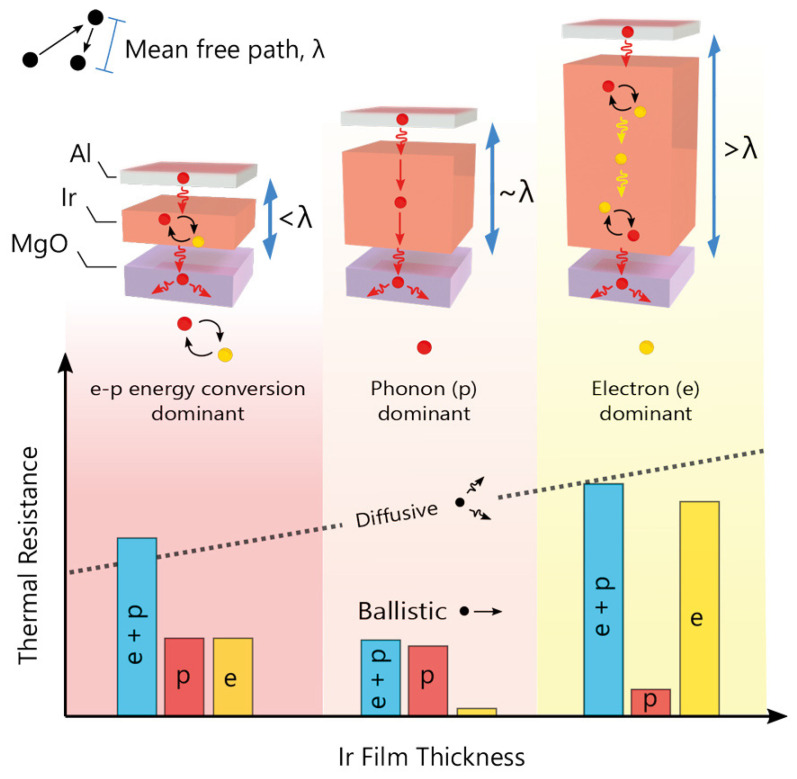
Schematic of the thermal resistance of the Al-Ir-MgO material stack [113].

Phonon transport mechanisms in NMMCs have been the subject of several recent studies, suggesting a variety of mechanisms. The electron–phonon coupling mechanism is important in the thermal transport of NMMCs, and it can be a mechanism for heat transfer at metal/non-metal interfaces [109,110]. Also, inelastic phonon transport mechanisms have been predicted to exist at interfaces between very dissimilar materials where the transmitted phonons do not have the same frequency as the incident phonons. A recent study observed inelastic phonon transport across atomically sharp metal/semiconductor interfaces [111]. Another mechanism is known as phonon-dominated energy transport, and it is especially dominant in pure metallic heterostructures [112]. The mechanism of phonon conduction can provide a heat transport mechanism even for metallic layers in metal/semiconductor heterostructures where the thickness of the metal layers is of the order of a few nanometers. Thermal conduction through phonons has also been shown to play an important role in heterostructures composed solely of metallic layers. A great deal of knowledge about the mechanisms underlying heat conduction in this context has been gained from studying heterostructures or multilayer composites made of metal and semiconductors. These heterostructures consist of semiconducting layers sandwiched between layers of a thin metallic material. In semiconductors, unbound electrons are absent, in contrast to metals, where thermal (and electrical) conduction is commonly attributed to the migration of free electrons, which are electrons that live within the metal lattice and are not bound to the atomic nuclei. In this instance, the migration of phonons—collective vibrations of the atoms in the semiconductor’s crystal structure—explains heat conduction. In this regard, Figure 12 shows a schematic of the thermal resistance of the Al-Ir-MgO material stack [113].

**Figure 13:**
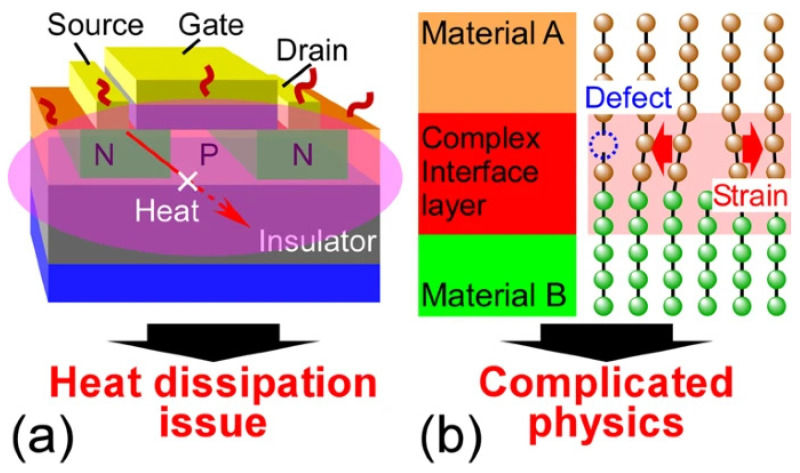
Thermal transport physics schematics across the interface. (**a**) Heat dissipation in a large-scale integrated circuit and (**b**) phonon transport at the interface of materials A and B [115].

As another mechanism, phonon scattering determines phonon transport behaviors in NMMCs. When the structural feature size is much greater than the phonon mean free path, three-phonon Umklapp scattering is dominant at high temperatures, and the heat transfer follows the classical thermal diffusion regime. When the structural feature size decreases to less than the phonon mean free path, phonon boundary scattering dominates [114]. Interfaces complicate the understanding of heat conduction at the nanoscale level. Therefore, in recent decades, academic research on interfacial nanostructured materials has been intensively carried out to elucidate their mechanism and control their thermal conductivity in various scientific fields, such as thermoelectric phononics. It should be noted that the dissipation of heat in nanoscale electronic devices has become a critical problem due to their high interface density, which hinders heat conduction, such as the SiO_2_ layer/Si for a Si-based large-scale integrated circuit. Figure 13 illustrates the schematics of thermal transport physics across the interface [115].

## 5. Electrical NMMCs

NMMCs have unique electrical properties that differ from those of bulk materials. NMMCs exhibit electrical behavior due to the high density of free electrons at the interfaces between the layers. The electrical resistivity of NMMCs is affected by the layer thickness, interface quality, and composition of the layers. A study on Cu/W multilayered nanofilms found that the electrical resistivity of the films decreases as the periodic thickness of the multilayers increases [106]. They can also exhibit magnetoresistance, which is the change in electrical resistance in the presence of a magnetic field. This effect is due to the spin-dependent scattering of electrons at the interfaces between the layers. Furthermore, quantum confinement effects can occur in NMMCs, where the electrons are confined to the layers and exhibit discrete energy levels. This can lead to changes in the electrical properties of the multilayers [1].

The exceptional electrical properties of NMMCs make them attractive for use in electronic devices. The low electrical resistivity of these materials can lead to improved performance in electronic devices that require high electrical conductivity. Additionally, the large number of interfaces in NMMCs can lead to unique electronic properties, such as enhanced electron scattering and quantum confinement effects [116]. These properties can be exploited to create new types of electronic devices with improved performance characteristics. Overall, the electrical properties of NMMCs make them promising candidates for use in a wide range of electronic applications. Some examples of electronic devices that use NMMCs include magnetic sensors, magnetic recording media, transparent conductive coatings, solar cells, microelectromechanical systems (MEMS), field-effect transistors (FETs), thermoelectric devices, batteries and supercapacitors, hydrogen storage, etc. [1]. These are just a few examples of the many potential applications of NMMCs in electronic devices.

### 5.1. Conductivity in Multilayer Composites

Utilization of NMMCs can enhance the electrical conductivity of electronic devices. There are several methods for enhancing the conductivity of NMMCs [117]. Some of these methods include the following:

(i) **Increasing the number of interfaces:** the large number of interfaces in NMMCs can lead to enhanced electron scattering and improved electrical conductivity;
(ii) **Controlling the layer thickness:** the thickness of the individual layers in NMMs can be precisely controlled, which can lead to improved electrical conductivity [1];
(iii) **Using conductive nanomaterials:** conductive nanomaterials, such as silver nanowires, can be incorporated into NMMCs to enhance their electrical conductivity [117];
(iv) **Fine-tuning the sheet resistance:** the electrical conductivity of NMMCs can be fine-tuned by adjusting the number of deposition cycles [118].

### 5.2. Quantum Size Effects on Electrical Conductivity

The electrical conductivity of NMMCs can be affected by quantum size effects. These effects arise due to the confinement of electrons in the nanoscale layers, which can lead to changes in the electronic properties of the material. The conductivity of quantized multilayer metal films has been analyzed with an emphasis on scattering by rough interlayer interfaces [119]. The quantum size effect can lead to enhanced electron scattering and improved electrical conductivity in some cases [120]. However, the effect can also lead to reduced electrical conductivity in other cases [121]. The conductivity of metallic films and multilayers can be calculated using quantum and semiclassical approaches, which take into account the confinement of electrons in the nanoscale layers [120]. Overall, the quantum size effect can have a significant impact on the electrical conductivity of NMMCs, and it is an important consideration in the design and optimization of electronic devices that use these materials. 

## 6. Radiation Tolerance NMMCs

The search results show that metallic multilayers can have high radiation-tolerance properties. The radiation tolerance of metallic multilayer systems to heavy ion irradiation inducing high damage levels has been evaluated. Radiation tolerance in metallic multilayers is attributed to the high density of interfaces that can absorb radiation-induced defects [30]. The interfaces in nanostructured metallic multilayers, such as nanocrystalline grain boundaries or layer interfaces, can enhance radiation tolerance by serving as sinks for radiation-induced defects. The significance of high radiation tolerance in multilayer coatings lies in their potential applications in environments with high levels of radiation, such as nuclear reactors, space, and high-energy physics facilities. The ability of these coatings to withstand and mitigate the effects of radiation exposure is crucial for ensuring the longevity and performance of sensitive equipment and components in these environments. High radiation tolerance in multilayer coatings can lead to several benefits, including the following:

(i) **Enhanced durability:** coatings with high radiation tolerance can protect underlying materials and components from degradation and damage caused by radiation exposure, thereby extending their operational lifespan [29];
(ii) **Improved reliability:** by withstanding radiation, multilayer coatings can contribute to the reliability and stability of critical components, reducing the need for frequent maintenance and replacement;
(iii) **Radiation shielding:** high radiation-tolerant coatings can serve as effective barriers against harmful radiation, safeguarding sensitive instruments and equipment from its detrimental effects [30];
(iv) **Versatile applications:** these coatings can find applications in diverse fields, including aerospace, nuclear technology, medical devices, and particle accelerators, where exposure to radiation is a concern [30].

### 6.1. Radiation Tolerance in Multilayer Composites

Usually, size-dependent radiation damage can be found in all systems. For instance, He can be stored in extremely high concentrations in nanolayer composites. By encouraging the recombination of point defects of the opposite type, layer interfaces lessen lattice distortion, swelling, and accumulative defect density. Interfaces also significantly reduce radiation hardening. When designing radiation-tolerant nanolayer composites, the chemical stability of the interfaces is a crucial factor. Systems that are immiscible or partially miscible are more stable when exposed to He ions [30]. A study based on ion beam analysis (IBA) of a nanometric Cr/Ta multilayer coating demonstrated an extremely high radiation tolerance [29]. The study used IBA techniques, including RBS (Rutherford backscattering spectroscopy) and NRA (nuclear reaction analysis), to investigate the radiation tolerance of the coating. The study found that the nanometric Cr/Ta multilayer coating demonstrated an extremely high radiation tolerance. The study also used TEM to analyze the microstructure of the coating [29]. Another study investigated the mechanical and radiation responses of Al/Nb and Fe/W multilayers to plastic deformation and high-fluence He ion irradiation conditions [122]. The study found that the miscible FCC/BCC interface with a large positive heat of mixing is not stable during ion irradiation and that radiation hardening is more significant at smaller layer thicknesses [122]. 

### 6.2. Potential Applications of Radiation Tolerance in Nanoscale Multilayer Composites

Radiation-tolerant NMMCs have potential applications in various fields, including nuclear technology, space, and high-energy physics facilities. The exceptional properties of NMMCs, such as their high density of interfaces, make them attractive for developing innovative materials with tailored characteristics. Some potential applications of radiation-tolerant NMMCs include the following:

(i) **Radiation shielding:** NMMCs can serve as effective barriers against harmful radiation, safeguarding sensitive instruments and equipment from its detrimental effects [30];
(ii) **Nuclear technology:** radiation-tolerant NMMCs can be used in nuclear reactors and other nuclear technology applications to enhance the durability and reliability of critical components [30];
(iii) **Space:** the ability of radiation-tolerant NMMCs to withstand and mitigate the effects of radiation exposure is crucial for ensuring the longevity and performance of sensitive equipment and components in space;
(iv) **High-energy physics facilities:** radiation-tolerant NMMCs can be used in high-energy physics facilities to protect sensitive detectors and other equipment from radiation damage.

## 7. Conclusions 

In conclusion, NMMCs are a class of materials composed of alternating layers of metals at the nanoscale level. These materials exhibit unique properties that are distinct from their bulk counterparts due to the confinement of electrons and the increased surface-to-volume ratio. The large number of interfaces and the nanoscale layer thicknesses are the main factors responsible for these properties. The review of the literature highlights the use of nanomultilayer architectures as viable routes to overcome the challenges of designing and implementing new engineering materials at the nanoscale. The manuscript classifies NMMCs based on their properties, including magnetic, optical, thermal, electrical, and radiation tolerance properties. 

NMMCs demonstrate enhanced magnetic properties, such as increased saturation magnetization, coercivity, and Curie temperature, compared to their bulk counterparts. These improvements are attributed to the exchange coupling between the ferromagnetic layers and the confinement of magnetic moments within the nanoscale dimensions. In addition, NMMCs have been shown to have enhanced optical properties, including tunable optical absorption and emission spectra, improved optical transparency, and enhanced nonlinear optical effects. These properties are influenced by the size, shape, and composition of the nanoscale layers, as well as the interactions between the layers. NMMCs exhibit improved thermal management capabilities, such as enhanced thermal conductivity and reduced thermal expansion coefficients. These properties are crucial for applications in electronic devices, where efficient heat dissipation is essential. The manuscript discusses the role of the layered structure and the interface between the layers in determining the thermal properties of NMMCs. Furthermore, the manuscript explores the enhanced electrical properties of NMMCs, including increased electrical conductivity, reduced electrical resistance, and improved electron transport. These properties are influenced by the confinement of electrons within the nanoscale layers and the interactions between the layers. NMMCs demonstrate improved radiation tolerance properties, making them suitable for applications in harsh environments, such as radiation shielding, nuclear technology, space, and high-energy physics facilities. The manuscript discusses the role of the layered structure and the interface between the layers in mitigating the effects of radiation damage, such as the formation of defects and the accumulation of impurities.

It should be noted that the development of metallic multilayers at the nanoscale, as seen in NMMCs, has evolved with synthesis technology and potential applications. Innovations in physical vapor deposition technologies, in particular magnetron sputtering, have enabled the synthesis of metallic-based structures with nanoscaled layer dimensions as small as one-to-two monolayers. The parameters used in the deposition process are paramount to the formation of these small layer dimensions and the stability of the structure. In summary, NMMCs have the potential to revolutionize materials science and engineering, and their exceptional properties make them attractive for a wide range of applications in various fields.

### Future Directions

The study of nanoscale metallic multilayer composites is a rapidly evolving field with significant implications for future research and technological advancements. These materials possess a unique combination of enhanced magnetic, optical, and radiation tolerance properties, making them highly valuable for a wide range of applications. The future directions of high-performance nanoscale metallic multilayer composites, encompassing magnetic, optical, radiation tolerance, thermal, and electrical properties, are poised for significant advancements and applications. The enhanced multifaceted properties of nanoscale metallic multilayer composites open up a vast array of potential applications. The findings presented in this study lay a solid foundation for future research in the field of nanoscale metallic multilayer composites. The potential future directions and areas of focus for these materials include the following:

(i) **Advanced synthesis methods:** continued research and development of advanced synthesis methods, such as physical vapor deposition technologies like magnetron sputtering, will enable the precise control and fabrication of metallic-based structures with nanoscaled layer dimensions.
(ii) **Enhanced material behaviors:** further exploration of the enhanced material behaviors exhibited by NMMCs, with a focus on tailoring their properties to meet specific application requirements in diverse fields.
(iii) **Multifunctional coatings:** advancement in the development of multifunctional coatings, particularly in the optical domain, leveraging the promising attributes of NMMCs for durable and transparent multifunctional coatings.
(iv) **Radiation tolerance studies:** continued research into the radiation tolerance properties of NMMCs, with a focus on understanding and enhancing their ability to withstand radiation damage, particularly in applications related to nuclear technology, space, and high-energy physics facilities.
(v) **Mechanical and thermal properties:** further investigations into the mechanical, thermal conductivity, and thermal stability properties of NMMCs are needed to expand their potential applications in areas requiring high-strength and thermally stable materials.
(vi) **Characterization techniques:** advancements in characterization techniques, such as X-rays, enable comprehensive analysis and understanding of the properties and behaviors of NMMCs.

In summary, the future directions of high-performance nanoscale metallic multilayer composites are poised to drive innovations in materials science and engineering, with a focus on advanced synthesis methods, multifunctional coatings, radiation tolerance studies, and comprehensive characterization techniques to unlock their full potential across various applications and industries. The findings of this study have the potential to serve as a foundation for future research and technological advancements. By leveraging the unique capabilities of these materials, researchers and engineers can drive progress in a wide range of fields, from electronics and energy to biomedical and defense applications. 

## Data Availability

All data generated or analyzed during this study are included in this published article.
